# Probing the Effects of Multisite Mutations in the Lipoic Acid Region of the BCOADC-E2 Protein

**DOI:** 10.3390/ijms252413677

**Published:** 2024-12-21

**Authors:** Jinjun Wang, Mingliang Yang, Huixian Wei, Wang Miao, Shiyu Li, Xinru Gao

**Affiliations:** 1 Key Laboratory of Arable Land Quality Monitoring and Evaluation, Ministry of Agriculture and Rural Affairs, Yangzhou University, Yangzhou 225009, China; 2 College of Environmental Science and Engineering, Yangzhou University, Yangzhou 225127, China; yasmine0787@163.com (M.Y.); weiii_hx@163.com (H.W.); miaowang_0322@163.com (W.M.); shirleyleenn@163.com (S.L.); gxr0207@163.com (X.G.)

**Keywords:** BCOADC-E2 protein, amino acid class, ELISA, SDSL-EPR technique

## Abstract

Primary biliary cholangitis (PBC) is a chronic disease, the prevalence of which has been increasing in recent years. And the prevalence of patients who test negative with existing diagnostic techniques remains high. It was found that the antigenic BCOADC-E2 protein could detect patients with a negative original test. And experiments revealed that the lipoyl domain of BCOADC-E2 plays an important role. The present study was carried out to verify the necessity of maintaining the folding conformation of the lipoyl β-sheet of the protein in the lipoyl domain during the recognition of the BCOADC-E2 protein and the importance of the glutamic acid and isoleucine residues at position 4 and position 13, respectively. In order to search for a new pathway for the pre-detection of patients with PBC, firstly, the mutant proteins were subjected to an enzyme-linked immunosorbent assay (ELISA) with serum. Then, MTSSL spin tags were positioned at specific sites of the Cys mutant and reacted with serum samples from PBC patients and controls, and EPR spectroscopic data were measured. The multiple mutant proteins all reacted less specifically with the serum than the wild-type protein in the ELISA; the spectra measured for the pGEX-BCKD-E4A-I13A mutant were severely broadened, and the compactness at the conformational position of the lipoyl β-sheet structural conformation of the proteins of amino acids 4 and 13 remained unchanged. The EPR spectral data validate the importance of the glutamate and isoleucine residues at position 4 and position 13 and their necessity in the maintenance of the lipoyl β-sheet structural conformation of proteins in the lipoyl domain in anti-BCOADC-E2 recognition.

## 1. Introduction

Primary biliary cholangitis (PBC) is an autoimmune liver disease [1,2]. It is accompanied by the destruction of intrahepatic bile ducts with inflammatory scarring, which ultimately leads to cholestasis and hepatic fibrosis [3] and may progress to cirrhosis and liver failure [4,5,6]. Both the PDC-E2 and BCOADC-E2 proteins are proteins that can be recognized with anti-mitochondrial antibodies (AMAs) [7]. Currently, the commercially available antigenic agents used in patients with PBC recognize the PDC-E2 protein. Approximately 5–10% of patients tested have negative AMA tests [8]. AMA-negative PBC cholangitis is associated with similar overall or liver-related mortality compared to AMA-positive disease [9]. In clinical practice, it has been found that the serum of approximately 4% to 13% of PBC patients does not recognize PDC-E2 but can recognize BCOADC-E2 [10,11]. Thus, BCOADC-E2 with AMA recognition is essential for the detection of PBC-negative patients [8,11,12]. This facilitates the improvement in serologic diagnostic accuracy in patients with PBC [11,12,13]. It has been shown that alanine substitutions in amino acids located near the active center of the inner lipoyl domain (ILD) of the BCOADC-E2 protein reduce the degree of specific reactive binding of the protein to AMA-M2 isoforms. The role of a single amino acid in antibody recognition depends on the sequence context in the antigenic domain [14]. This study mutates key amino acid residues of this structural domain in a multisite combination to investigate the importance of the critical amino acids of the thioctyl structural domain within the BCOADC-E2 protein and the integrity of this structural domain for AMA-M2 recognition. Based on the successful homology modelling, it was shown that the key amino acids at positions 4, 5, 12, and 13 of the lipoyl domain of the BCKD protein (both BCOADC-E2 proteins used in the experimental procedure; hereinafter replaced by the abbreviation pGEX-BCKD) showed a two-by-two approximate symmetric distribution around the active center, lysine (K). On this basis, the four key amino acids were mutated to explore the importance of the anti-BCOADC-E2 serologic reactivity and β-folded conformational integrity of the mutant protein and the importance of the hydrophobic arm of the isoleucine residue in maintaining the β-folded conformation of the lipoyl domain.

Directed spin labelling–electron paramagnetic resonance (SDSL-EPR) is a technique in which an amino acid site of a protein is mutated to a cysteine (Cys). Then, the unpaired electrons in the paramagnetic marker are probed using the EPR technique to obtain the spectral information of the specific site, so as to analyze the conformational features of the protein-specific site [15,16]. SDSL-EPR has become an important tool for monitoring the sequence-specific structure and protein dynamics [17], which is an important method to probe the conformation of the BCOADC-E2 protein in this paper.

## 2. Results

### 2.1. Construction and Expression of Multi-Mutant Plasmids

#### Sequencing Results of Multi-Mutant Plasmid

**Figure 1:**
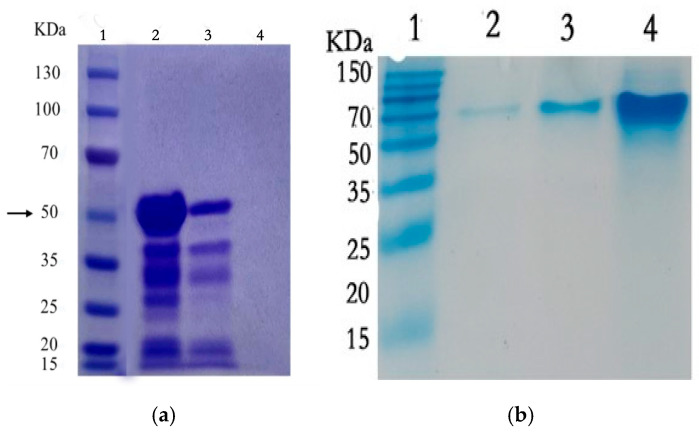
(**a**) Successful protein size map of mutant protein pGEX-BCKD-V5A-T12A-I13A expression. ‘→’ points to the molecular weight of the target protein. Lane 1: two-color pre-stained protein marker; lanes 2, 3: triple mutant expressed protein; lane 4: blank. (**b**) Electropherogram of the wild-type protein pGEX-BCKD. Lane 1: two-color pre-stained protein marker; lanes 2, 3, and 4: pGEX-BCKD.

A total of nine multi-mutant plasmids were extracted. And after sequencing, we could see that bases 4, 5, 12, and 13 of the design sites of the four mutant plasmids were successfully mutated to alanine, and the rest of the amino acids were kept intact. The recombinant plasmid was successfully constructed. After SDS-PAGE and staining and destaining with Kaomas Brilliant Blue R250, the protein gel map was obtained (taking pGEX-BCKD-V5A-T12A-I13A as an example). It was observed that the protein band was at 50 KDa (Figure 1), which was consistent with the expected size of the protein. The remaining protein bands with smaller molecular weights are formed from small amounts of target protein degraded by proteases. The protein expression was successful.

### 2.2. Serum Immunoreactivity of Polymorphic Proteins Against PBC

**Figure 2:**
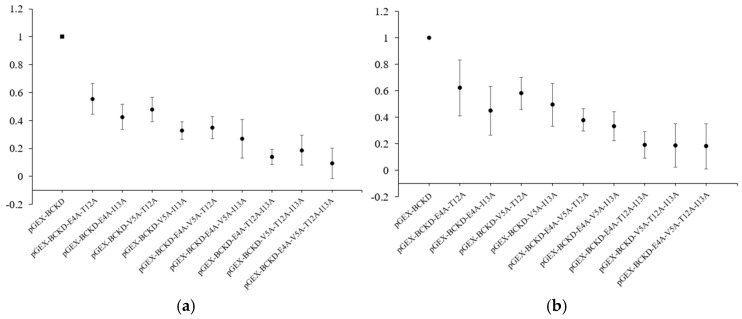
Average relative binding ratios of multi-mutant proteins relative to the wild-type protein: (**a**) primary antibody dilution ratio 1:4000; (**b**) primary antibody dilution ratio 1:16,000.

The degree of serum-specific reactivity of multi-mutant proteins with the serum was lower than that of wild-type proteins in both ELISAs. The average OD450 values of the multi-mutant proteins showed a higher to lower degree of fold response to the wild proteins in the following order (Table 1): double mutant proteins > triple mutant proteins > quadruple mutant proteins. The degree of reduced specific response showed a positive correlation with the number of four key amino acid mutation sites (Figure 2).

**Figure 3:**
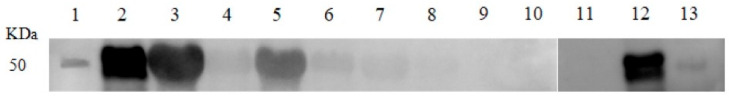
Western Blot developments. Lanes 1, 13: two-color pre-stained protein marker; lanes 2, 12: pGEX-BCKD; lane 3: pGEX-BCKD-E4A-T12; lane 4: pGEX-BCKD-E4A-I13A; lane 5: pGEX-BCKD-V5A-T12A; lane 6: pGEX-BCKD-V5A-I13A; lane 7: pGEX-BCKD-E4A-V5A-T12A; lane 8: pGEX-BCKD-E4A-V5A-I13A; lane 9: pGEX-BCKD-E4A-T12A-I13A; lane 10: pGEX-BCKD-V5A-T12A-I13A; lane 11: GEX-BCKD-E4A-V5A-T12A-I13A. Note: the first antiserum (LX) is diluted at a ratio of 1:1000, and the second antiserum (HRP goat anti-human IgG) is diluted at a ratio of 1:10,000.

The Western Blot (Figure 3) showed that the specific reaction bands of the multi-mutant proteins were all located at 50 KDa, and the protein sizes were correct. The color of the bands in lanes 2–10 was lighter than that in No. 1. And the degree of the specific reaction of the nine multi-mutant proteins was lower than that of the wild-type protein, i.e., the combinations of multi-mutations of any amino acids in positions 4, 5, 12, and 13 decreased the BCOADC-E2 protein’s ability to specifically recognize AMA-M2. And among the most responsive double mutations, pGEX-BCKD-E4A-T12A (lane 2) showed the highest degree of specificity, followed by pGEX-BCKD-V5A-T12A (lane 4). Lastly, pGEX-BCKD-V5A-I13A (lane 3) and pGEX-BCKD-V5A-I13A (lane 5) had weak protein-specific responses.

Combined with the graphs, it can be seen that the mutant proteins produced by the substitution of isoleucine at position 13 by alanine all show a substantial decrease in the degree of serum-specific response. This indicates the importance of the isoleucine residue at position 13 for the recognition of AMA-M2 by the BCOADC-E2 protein.

### 2.3. Results of EPR Spectroscopy

The sequencing results of the Cys protein mutant plasmid: on the basis of pGEX-BCKD-E4A-I13A, PCR technology introduced the Cys mutation successfully, and Glu4, Ile13, and Glu4/Ile13 mutants were constructed successfully.

**Figure 4:**
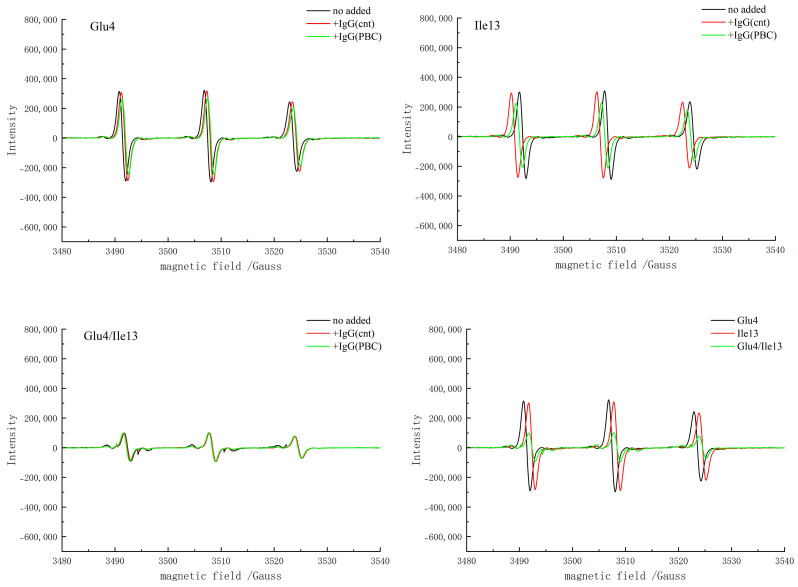
X-band EPR spectra of the lipoyl domain in vitro in the spin-labelled Cys-containing substitution mutation.

X-band scanning was performed using the SDSL-EPR technique. The spectra of mutants Glu4, Ile13, and Glu4/Ile13 were compared and analyzed after reacting with the blank, normal human serum, and PBC patient serum, respectively. The results showed that the positions of the endolipoyl structural domains Glu4 and Ile13 should be approximately opposite and close to each other. When the distance between the spin labels was within 2 nm, significant dipole coupling was detected, leading to spectral broadening, and the spectral broadening of the Glu4/Ile13 mutant was significantly increased compared with that of Glu4 and Ile13. This indicates the presence of dipole–dipole coupling in Glu4/Ile13 (Figure 4). That is, in the case of the Glu4/Ile13 double mutant, the conformational closeness of the secondary β-sheet domains of the amino acid proteins of amino acids 4 and 13 remained unchanged (1–1.5 nm). Consistent with expectations, PBC patient sera bound to this mutant to a lesser extent. The addition of this antibody to double spin-labelled proteins did not significantly alter the complex spectra.

High affinity between serum molecules leads to enhanced intermolecular interactions. This leads to an enhanced restricted spin motion of electrons in the spin-labelled material, resulting in a wider EPR spectrum. However, only with the addition of PBC patient serum, moderate broadening was observed for Glu4 and Ile13. Ile13 broadening occurred to a greater extent, and no significant effect was observed for the control serum.

## 3. Discussion

PBC is a chronic cholestatic liver disease [18], characterized by chronic non-suppurative destructive cholangitis and usually affecting middle-aged women [19,20,21]. The disease is relatively rare with a single treatment [22] and limited therapeutic agents [23]. The common symptoms are fatigue and itching [24], which have a significant impact on long-term quality of life [25]. The disease may be asymptomatic in its early stages, leading to misdiagnosis which can affect the condition [26,27,28]. Therefore, early diagnosis is important for PBC. Previous studies have shown that amino acids at position 5 and 13 of the BCOADC-E2 protein are the key amino acids that affect its specific response with AMA-M2. In-depth study of the BCOCAD-E2 protein is important for the resolution and prevention of PBC.

In this study, the functional structure of the BCOADC-E2 protein and the importance of sites 4 and 13 for maintaining the β-folded conformation of the protein were explored. The antigenic epitope of the BCOADC-E2 protein is located in the lipoyl domain, which is the active center of the protein. It has become a hotspot to study the effect of amino acids around the active center of the protein on protein function [29]. The amino acid residues around the lysine of the active center of the BCOADC-E2 protein were sequentially replaced by alanine scanning for targeted mutagenesis [30,31,32] and then analyzed by the SDSL-EPR technique. The results showed that all combinations of multi-mutant proteins reduced the degree of specific response to the serum and were positively correlated with the number of mutations in the four critical amino acids. A greater degree of decline was observed in both ELISAs and Western Blot chromatograms after the substitution of site 13, and the expressed recombinant proteins showed a reduced sensitivity to reacting with AMA-M2, which is beneficial for the early diagnosis of PBC. The spectrum of the mutant pGEX-BCKD-E4A-I13A (Glu4/Ile13) with the most pronounced decrease was later severely broadened by EPR, indicating a strong dipolar coupling between the spin tags, unchanged tightness of the protein β-sheet conformation, and a certain effect of AMA-M2 in the sera of patients with PBC on the dynamics of the spin tags, consistent with the pGEX-BCKD-E4A-I13A mutation, further suggesting that site 13 has an important role in anti-BCOADC-E2 recognition, which is consistent with our previous experimental results [33]. The recognition of key amino acids at positions 4 and 13 might contribute to the development of novel vaccines [34,35,36] and specific immunotherapy for the PBC disease [36].

With the antigenic identification and cloning of AMA/M2-specific reactions, we have a better understanding of PBC. However, the role of AMA in the pathogenesis of PBC is unclear, and there is no consensus on the value of AMA in the early diagnosis and prognosis of PBC. The literature data show different outcomes of AMA-positive patients that are decidedly environmental; 0.07–9.9% of healthy individuals can present with AMA positivity, and only 0.73% of AMA-positive Japanese patients present with PBC symptoms. And whether AMA can predict the occurrence of PBC is still highly controversial [37,38,39,40]. Can the power of amino acid deprivation be harnessed as a novel strategy in the treatment of PBC [41]? This needs to be further explored.

In summary, in this study, using protein mutagenesis and functional validation, we verified that the degree of BCOADC-E2 protein binding to AMA-M2 is importantly related to sites 4 and 13 in the lipoyl domain. We further confirmed this using the EPR technique. This suggests that two key amino acids, glutamic acid at position 4 and isoleucine residue at position 13, offer the possibility of detecting PBC patients in the future. It provides a lesson and reference for accurate early PBC diagnosis. And the maintenance of the folded conformation of the β-sheet domain of the lipoyl domain protein in anti-BCOADC-E2 recognition is necessary for the accurate diagnosis of early PBC.

## 4. Materials and Methods

### 4.1. Sequencing of Multi-Mutant Plasmid

**Figure 5:**
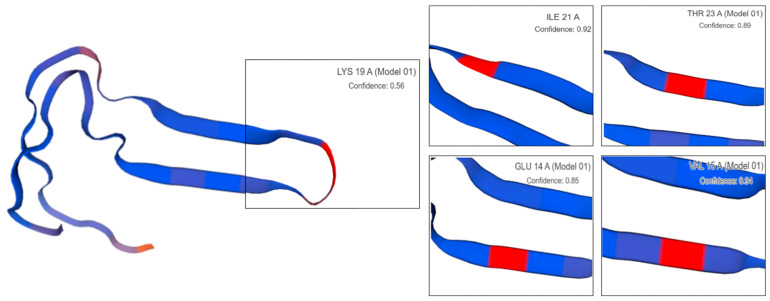
Homology modelling of the active center of the thioctyl structure domain within the BCOADC-E2 protein.

The four key amino acids at positions 4, 5, 12, and 13 of the BCKD protein show a two-by-two approximately symmetric distribution around the active center lysine (K) (Figure 5). The primer design tool used was Quick Change (https://www.agilent.com.cn/store/primerDesignProgram.jsp, accessed on 5 April 2022). The mutation kit used was the Fast Mutagenesis System kit. Targeted mutagenesis was performed using pGEX-BCKD as a template. The 4 key amino acids before and after K, the active center of the protein’s thiooctanoyl structural domain, were multi-mutated to neutral alanine. There were a total of 9 multi-mutant plasmids (as shown in Table 2). The plasmids were sent to be sequenced by Shanghai Sangong Biotechnology Co. (Shanghai, China).

### 4.2. Purification of Multi-Mutant Proteins

#### 4.2.1. Protein Expression

Correct sequencing mutants were cultured on LB plates containing ampicillin (100 mg/mL) and then cultured at a 1:50 ratio until OD600 = 0.6–0.8, induced by adding IPTG (final concentration of 0.5 mM), with shaking cultivation at 24 °C, 150 rpm, overnight.

#### 4.2.2. Separation and Purification

Firstly, the GSTrapTM chromatography column was used to wash out the heterogeneous proteins and remove the GST tags. Then, they were incubated in the chromatography column with 1000 U thrombin solution diluted to a total of 2 mL at 25 °C, and then the GSTrapTM chromatography column was connected with the HiTrapTM Benzamidine FF pre-packed column (Sytiva Biotech Co., Ltd., Jiaxing, China) to elute the desired proteins.

### 4.3. Immunoreactivity of Multi-Mutant Proteins Against PBC Sera

#### 4.3.1. ELISA Test

The nine purified mutant proteins and the wild-type protein pGEX-BCKD (retained in the laboratory for the recombinant BCOADC-E2 protein) were used as antigens. Nine PBC serum samples were used as primary antibodies (dilution ratio of 1:4000). The primary antibody serum samples were LX04881, LX04889, LX04809, LX04872, LX04921, LX04916, LX04924, LX04946, and LX04887 (provided by Nanjing Medical University, Nanjing, China). And rabbit anti-human IgG H&L (HRP) was used as a secondary antibody (dilution ratio of 1:16,000, Beijing Bosun Biotechnology Co., Ltd., Beijing, China) to detect the degree of specificity of the mutant proteins in response to AMA-M2.

The experiments were set up with three parallels and one blank for each group, and a total of two groups were used to perform the indirect ELISA experiments [42]. The specific reaction of 10 proteins to 9 serum samples of PBC patients was measured.

#### 4.3.2. Protein Blotting Method

SDS-PAGE gels were made by referring to the Bio-Rad TGX Stain-free kit instructions (Shanghai, China). The concentration of each protein was uniformly diluted to 19 mg/mL for up-sampling. The negative controls were blank. In total, 10 µL of each mutant protein with the same concentration of 19 mg/mL was used. The primary antiserum (LX04872) was diluted at a ratio of 1:1000, and the second antiserum (HRP goat anti-human IgG) was diluted at a ratio of 1:10,000. The antibodies were the same as those detected by ELISA. The wild-type protein pGEX-BCKD, nine mutant proteins of the experimental group, and Elution Buffer of the blank group were subjected to SDS-PAGE.

### 4.4. EPR

#### 4.4.1. Cys Mutation Model Construction

To further validate the importance of the glutamic acid at position 4 and the isoleucine residue at position 13 and the necessity of the folding conformational integrity of the β-sheet structural domain of the lipoyl domain in the recognition of anti-BCOADC-E2, a new study was carried out. We selected the double mutant pGEX-BCKD-E4A-I13A for mutagenesis. Its specific response to AMA was lower than that of the wild type (pGEX-BCKD) and higher than that of the triple mutant. Three mutants were obtained by mutating amino acids at positions 4 and 13 with Cys. Spin tags were then localized to the three mutants, followed by PCR. The replacement mutants were named Glu4, Ile13, and Glu4/Ile13 (Table 3); PCR was conducted using the Fast Mutagenesis System kit (Beijing All Style Gold Biotechnology Co., Ltd., Beijing, China), followed by protein purification and strain preservation.

#### 4.4.2. EPR Spectroscopy Detection

The dissolved MTSSL solution was mixed with Cys purified protein solution and incubated overnight at 4 °C [43]. The final concentration of MTSSL was about 500 mM. After labelling, the protein solution was concentrated to approximately 1 mg/mL. Then, 25 μL of concentrated protein solution was taken and mixed with 25 μL of normal human serum and PBC patient serum for reaction, capillary inhalation of about 20 μL of post-reaction samples, Rubbermaid sealing, and EPR measurements in a paramagnetic resonance spectrometer. Detection conditions: 100 G electric field; modulated wave amplitude, 1 G; microwave power, 20 mW; central magnetic field, 3503.37 G; and scan time, 80 s.

## Figures and Tables

**Table 1:** ELISA OD450 values of multi-mutant proteins relative to wild-type proteins.

Number	Designation	Primary Antibody Dilution Ratio 1:4000	Primary Antibody Dilution Ratio 1:16,000
	pGEX-BCKD	1.000 ± 0.000	1.000 ± 0.000
A	pGEX-BCKD-E4A-T12A	0.621 ± 0.210	0.556 ± 0.111
B	pGEX-BCKD-E4A-I13A	0.447 ± 0.182	0.426 ± 0.091
C	pGEX-BCKD-V5A-T12A	0.579 ± 0.119	0.479 ± 0.088
D	pGEX-BCKD-V5A-I13A	0.494 ± 0.163	0.328 ± 0.064
E	pGEX-BCKD-E4A-V5A-T12A	0.378 ± 0.083	0.351 ± 0.079
F	pGEX-BCKD-E4A-V5A-I13A	0.332 ± 0.108	0.269 ± 0.138
G	pGEX-BCKD-E4A-T12A-I13A	0.191 ± 0.100	0.139 ± 0.054
H	pGEX-BCKD-V5A-T12A-I13A	0.186 ± 0.163	0.187 ± 0.107
I	pGEX-BCKD-E4A-V5A-T12A-I13A	0.180 ± 0.171	0.096 ± 0.109

**Table 2:** Control table before and after amino acid mutation.

Mutation Type	Number	Designation	Mutagenic Gene	Final Sequence
Double mutation	A	pGEX-BCKD-E4A-T12A	ACT → GCT	SICAVQSDKASVAITSR
	B	pGEX-BCKD-E4A-I13A	ATC → GCC	SICAVQSDKASVTATSR
	C	pGEX-BCKD-V5A-T12A	ACT → GCT	SICEAQSDKASVAITSR
	D	pGEX-BCKD-V5A-I13A	ATC → GCC	SICEAQSDKASVTATSR
Triple mutation	E	pGEX-BCKD-E4A-V5A-T12A	GAA → GCA	SICAAQSDKASVAITSR
	F	pGEX-BCKD-E4A-V5A-I13A	GAA → GCA	SICAAQSDKASVTATSR
	G	pGEX-BCKD-E4A-T12A-I13A	ACT → GCT	SICAVQSDKASVAATSR
	H	pGEX-BCKD-V5A-T12A-I13A	ACT → GCT	SICEAQSDKASVAATSR
Tetrad mutation	I	pGEX-BCKD-E4A-V5A-T12A-I13A	ATC → GCC	SICAAQSDKASVAATSR

Note: ‘→’ denotes mutant amino acids.

**Table 3:** Sequence list of Cys substitution mutants.

Designation	Sequences
Glu4	SICAVQSDKASVTATSR
Ile13	SISAVQSDKASVTACSR
Glu4/Ile13	SICAVQSDKASVTACSR

## Data Availability

The data that support the finding of this study are available from the corresponding author upon reasonable request.
